# Medical negligence: Coverage of the profession, duties, ethics, case law, and enlightened defense - A legal perspective

**DOI:** 10.4103/0970-1591.56206

**Published:** 2009

**Authors:** M. S. Pandit, Shobha Pandit

**Affiliations:** Corporate Advocates, D- 29, 5^th^ Floor, Mantri Kishore Park, Bhosale Nagar, Pune 411 007, India

**Keywords:** Error of judgment, medical negligence, prior informed consent

## Abstract

A patient approaching a doctor expects medical treatment with all the knowledge and skill that the doctor possesses to bring relief to his medical problem. The relationship takes the shape of a contract retaining the essential elements of tort. A doctor owes certain duties to his patient and a breach of any of these duties gives a cause of action for negligence against the doctor. The doctor has a duty to obtain prior informed consent from the patient before carrying out diagnostic tests and therapeutic management. The services of the doctors are covered under the provisions of the Consumer Protection Act, 1986 and a patient can seek redressal of grievances from the Consumer Courts. Case laws are an important source of law in adjudicating various issues of negligence arising out of medical treatment.

## WHAT IS MEDICAL NEGLIGENCE?

The medical profession is considered a noble profession because it helps in preserving life. We believe life is God given. Thus, a doctor figures in the scheme of God as he stands to carry out His command. A patient generally approaches a doctor/hospital based on his/its reputation. Expectations of a patient are two-fold: doctors and hospitals are expected to provide medical treatment with all the knowledge and skill at their command and secondly they will not do anything to harm the patient in any manner either because of their negligence, carelessness, or reckless attitude of their staff. Though a doctor may not be in a position to save his patient's life at all times, he is expected to use his special knowledge and skill in the most appropriate manner keeping in mind the interest of the patient who has entrusted his life to him. Therefore, it is expected that a doctor carry out necessary investigation or seeks a report from the patient. Furthermore, unless it is an emergency, he obtains informed consent of the patient before proceeding with any major treatment, surgical operation, or even invasive investigation. Failure of a doctor and hospital to discharge this obligation is essentially a tortious liability. A tort is a civil wrong (*right in rem*) as against a contractual obligation (*right in personam*) – a breach that attracts judicial intervention by way of awarding damages. Thus, a patient's right to receive medical attention from doctors and hospitals is essentially a civil right. The relationship takes the shape of a contract to some extent because of informed consent, payment of fee, and performance of surgery/providing treatment, etc. while retaining essential elements of tort.

In the case of Dr. Laxman Balkrishna Joshi *vs*. Dr. Trimbark Babu Godbole and Anr., AIR 1969 SC 128 and A.S.Mittal v. State of U.P., AIR 1989 SC 1570, it was laid down that when a doctor is consulted by a patient, the doctor owes to his patient certain duties which are: (a) duty of care in deciding whether to undertake the case, (b) duty of care in deciding what treatment to give, and (c) duty of care in the administration of that treatment. A breach of any of the above duties may give a cause of action for negligence and the patient may on that basis recover damages from his doctor. In the aforementioned case, the apex court interalia observed that negligence has many manifestations – it may be active negligence, collateral negligence, comparative negligence, concurrent negligence, continued negligence, criminal negligence, gross negligence, hazardous negligence, active and passive negligence, willful or reckless negligence, or negligence per se. Black's Law Dictionary defines negligence per se as “conduct, whether of action or omission, which may be declared and treated as negligence without any argument or proof as to the particular surrounding circumstances, either because it is in violation of statute or valid Municipal ordinance or because it is so palpably opposed to the dictates of common prudence that it can be said without hesitation or doubt that no careful person would have been guilty of it. As a general rule, the violation of a public duty, enjoined by law for the protection of person or property, so constitutes.”

### Negligence per se

While deliberating on the absence of basic qualifications of a homeopathic doctor to practice allopathy in Poonam *Verma vs*. Ashwin Patel and Ors. (1996) 4 SCC 322, the Supreme Court held that a person who does not have knowledge of a particular system of medicine but practices in that system is a quack. Where a person is guilty of negligence per se, no further proof is needed.

### Duty on the part of a hospital and doctor to obtain prior consent of a patient

There exists a duty to obtain prior consent (with respect to living patients) for the purpose of diagnosis, treatment, organ transplant, research purposes, disclosure of medical records, and teaching and medico-legal purposes. With respect to the dead in regard to pathological post mortem, medico-legal post mortem, organ transplant (for legal heirs), and for disclosure of medical record, it is important that informed consent of the patient is obtained. Consent can be given in the following ways:

- Express Consent: It may be oral or in writing. Though both these categories of consents are of equal value, written consent can be considered as superior because of its evidential value.
- Implied Consent: Implied consent may be implied by patient's conduct.
- Tacit Consent: Tacit consent means implied consent understood without being stated.
- Surrogate consent: This consent is given by family members. Generally, courts have held that consent of family members with the written approval of 2 physicians sufficiently protects a patient's interest.
- Advance consent, proxy consent, and presumed consent are also used. While the term advance consent is the consent given by patient in advance, proxy consent indicates consent given by an authorized person. As mentioned earlier, informed consent obtained after explaining all possible risks and side effects is superior to all other forms of consent.

### The importance of obtaining informed consent

In the case of Samira Kohli *vs*. Dr. Prabha Manchanda and Ors. I (2008) CPJ 56 (SC), the apex court held that consent given for diagnostic and operative laparoscopy and “laporotomy if needed” does not amount to consent for a total hysterectomy with bilateral salpingo opherectomy. The appellant was neither a minor nor mentally challenged or incapacitated. As the patient was a competent adult, there was no question of someone else giving consent on her behalf. The appellant was temporarily unconscious under anesthesia, and as there was no emergency. The respondent should have waited until the appellant regained consciousness and gave proper consent. The question of taking the patient's mother's consent does not arise in the absence of emergency. Consent given by her mother is not a valid or real consent. The question was not about the correctness of the decision to remove reproductive organs but failure to obtain consent for removal of the reproductive organs as performance of surgery without taking consent amounts to an unauthorized invasion and interference with the appellant's body. The respondent was denied the entire fee charged for the surgery and was directed to pay Rs. 25000/- as compensation for the unauthorized surgery.

### Coverage of doctors and hospitals under CPA

In the case of the Indian Medical Association *vs*. V.P. Shanta and Ors., III (1995) CPJ 1 (SC), the Supreme Court finally decided on the issue of coverage of medical profession within the ambit of the Consumer Protection Act, 1986 so that all ambiguity on the subject was cleared. With this epoch making decision, doctors and hospitals became aware of the fact that as long as they have paid patients, all patients are consumers even if treatment is given free of charge. While the above mentioned apex court decision recognizes that a small percentage of patients may not respond to treatment, medical literature speaks of such failures despite all the proper care and proper treatment given by doctors and hospitals. Failure of family planning operations is a classic example. The apex court does not favor saddling medical men with *ex gratia* awards. Similarly, a in a few landmark decisions of the National Commission dealing with hospital death, the National Commission has recognized the possibility of hospital death despite there being no negligence.

## WHERE COMPENSATION WAS AWARDED

In this context, it may be recalled that in the case of the State of Haryana and Ors v. Smt. Santra, I (2000) CPJ 53 (SC) (by S. Saghir Ahmad and D.P.Wadhwa, JJ.), the Supreme Court in a Special Leave Petition upheld the claim for compensation where incomplete sterilization (family planning operation) was held to be defective in service. Smt Santra underwent a family planning operation related only to the right fallopian tube and the left fallopian tube was not touched, which indicates that complete sterilization operation was not performed. A poor laborer woman, who already had many children and had opted for sterilization, became pregnant and ultimately gave birth to a female child in spite of a sterilization operation that had obviously failed.

Claim for damages was based on the principle that if a person has committed civil wrong, he must pay compensation by way of damages to the person wronged. The apex court held: “Maintenance” would obviously include provision for food, clothing, residence, education of the children and medical attendance or treatment. The obligation to maintain besides being statutory in nature is also personal in the sense that it arises from the very existence of the relationship between a parent and the child. Claim for damages, on the contrary, is based on the principle that if a person has committed civil wrong, he must pay compensation by way of damages to the person wronged.

While elaborating on medical negligence, the apex court observed as follows (abridged): Negligence is a ‘tort’. Every doctor who enters into the medical profession has a duty to act with a reasonable degree of care and skill. This is what is known as ‘implied undertaking’ by a member of the medical profession that he would use a fair, reasonable and competent degree of skill. In the case of Bolam V. Friern Hospital Management Committee, (1957) 2 All ER 118, McNair, J. summed up the law as the following:

“The test is the standard of the ordinary skilled man exercising and professing to have that special skill. A man need not possess the highest expert skill: It is well established law that it is sufficient if he exercises the ordinary skill of an ordinary competent man exercising that particular art. In the case of a medical man, negligence means failure to act in accordance with the standards of reasonably competent medical men at the time. There may be one or more perfectly proper standards, and if he confirms with one of these proper standards, then he is not negligent.”

In the case of Spring Meadows Hospital and Anr. v Harjol Ahluwalia, 1998 4 SCC 39, a compensation of Rs. 5 lacs was awarded because of mental anguish caused to the parents of a child who became totally incapacitated for life in addition to a compensation of Rs. 12 lacs approx. awarded to the child. While the amount of Rs. 12 lacs was to be paid by insurer, the balance amount was to be paid by the hospital. Though the insurance company took a stand since the nurse who administered the adult dose of inj. Lariago to the child was not qualified, the apex court did not go into this issue while adjudicating negligence related proceeding. Therefore, it is important to keep in mind that doctors and hospitals should not only obtain a Professional Indemnity Insurance Policy, but also take care that nurses and other hospitals staff engaged by it are qualified.

## MEDICAL ETHICS AND THE TREATMENT OF ACCIDENT VICTIMS

In the case of Pravat Kumar Mukherjee *vs*. Ruby General Hospital and Ors, II(2005)CPJ35(NC), the National Commission delivered a landmark decision concerning treatment of an accident victim by the hospital. The brief facts of the case are as follows: the complainants are the parents of the deceased boy. They approached the National Commission for compensation and adequate relief. The case involves the unfortunate death of a young boy, Shri Sumanta Mukherjee, a student of second year B. Tech., Electrical Engineering. At Netaji Subhash Chandra Bose Engineering College on January 14, 2001 a bus from Calcutta Tramway Corporation crashed with the motorcycle driven by the deceased. Sumanta was conscious after the accident and was taken to the hospital about 1 km from the site of the accident. He was insured for Rs. 65,000/- under a Mediclaim Policy issued by the New India Assurance Co. Ltd. When he reached the hospital, the deceased was conscious and showed the Mediclaim certificate he was carrying in his wallet. He also assured that charges for treatment would be paid and treatment should be started. Acting on this promise, the hospital started treatment in its emergency room by giving moist oxygen, starting suction, and by administering injection Driphylline, Injection Lycotinx, and titanous toxoid. The respondents demanded an immediate payment of Rs. 15000/- and discontinued treatment as the amount was not deposited immediately though an assurance to pay the amount was made by the accompanying persons from the general public. Actually, the crowd collected Rs. 2000/- and the amount with the motorcycle of the patient and insurance receipt was offered. As the hospital was adamant and discontinued treatment after giving treatment for 45 minutes, the people from the crowd present were forced to take the patient to National Calcutta Medical College, which is about 7-8 km from the current hospital. The patient died on the way and was declared dead upon arrival at the National Calcutta Medical College.

The National Commission allowed the complaint and the Opponent Ruby Hospital was directed to pay Rs. 10 lakhs to the Complainant for mental pain agony. The Commission observed as follows: “This may serve the purpose of bringing about a qualitative change in the attitude of the hospitals of providing service to human beings as human beings. A human touch is necessary; that is their code of conduct; that is their duty and that is what is required to be implemented. In emergency or critical cases, let them discharge their duty/social obligation of rendering service without waiting for fee or for consent”. However, it remains to be seen whether the above award has brought in any attitudinal change in the medical fraternity.

An award was given on the following basis/grounds. While dealing with the contention that ‘no consideration paid’, ‘deceased or complainant not consumer’ National Commission observed as follows (abridged): “Not acceptable. Persons belonging to the poor class who are provided service free of charge are beneficiaries of service which is hired or availed of by the paying class. The status of an emergency or critically ill patient would be the same as people belonging to the poor class since both are not in a position to pay. Free services would also be services and the recipient would be the consumer under the Act. Since doctors started treatment on the deceased due to an emergency, that itself is availing of services, may it be free of cost or promised deferred payment. Expert evidence pointed out that discontinuance of treatment hastened the death of the patient, which itself is deficiency in service. Serious negligence and laxity on the part of the hospital by refusing admission and treatment facility to the youth who was almost in dying condition, defying all medical ethics and a gross violation of the Clinical Establishment rules and Act of 1950 as amended in 1998. How was a patient who was advised admission at ITU was allowed to leave the hospital for treatment elsewhere without signing any document or risk bond not shown? Withdrawal of treatment can not be justified on any ground. Deficiency is writ large.

Secondly, while dealing with the contention that there was no consent for treatment, the National Commission observed as follows (abridged): “Since emergency treatment is required to be given to a patient who was brought in seriously injured condition there was no question of waiting for consent. Consent is implicit in such cases. On the contrary, a surgeon who fails to perform an emergency operation must prove that the patient refused to undergo an operation not only at the initial stage but even after he was informed about the dangerous consequences of not undergoing the operation. Waiting for consent of a patient or a passer-by who brought the patient to the hospital is nothing but absurd and is apparent failure of duty on the part of doctor. Deficiency in service was proved and compensation was granted.

Maintainability of a consumer case when a Motor Accident Claims Tribunal (MACT) case is pending: The National Commission held that the MACT case is no bar to complaint under CP Act. Two causes are different and required to be decided by separate tribunals/forums. While the cause of action before MACT was rash and negligent driving, due to which the accident was caused, the cause of action against doctors and hospitals is for deficiency in rendering service – emergency treatment by the doctors or the hospital. Since both causes are separate and distinct, complaint is maintainable.

## THE IMPORTANCE OF CASE LAW

Jurisprudential principle of ‘stare decisis’ is based on a Latin phrase meaning to stand by decided cases; to uphold precedents; to maintain the positions laid down by higher courts earlier. One of the important characteristics of a good law is that the law should be definite, lucid, and unambiguous with the flexibility to relate to different situations, facts, and circumstances and that justice is done in accordance with law. Latin maxim ‘Stare decisis, et non quieta movere’ means it is best to adhere to decisions and not to disturb questions put at rest. The objective is to avoid confusion in the minds of the citizens as to what the law of the land is. As laid down in u.a 141 of the constitution of India, the law declared by the Supreme Court is binding in all courts. Furthermore, the Constitution of India provides that both the Supreme Court and High Courts of States are the courts of records. So far as the case law laid down by the National Commission and State Commission is concerned, they are followed by lower fora as a binding precedent though no specific provision has been made in the Consumer Protection Act, 1986. It is generally accepted that when a point of law is settled by a decision of a superior authority, it is not to be departed from. Change of a judge shall not affect the settled legal position. A new judge is not supposed to pronounce a new law but is expected to maintain and expound the old one. While this appears to restrict the elbow room of new judges to interpret the law when there is a settled legal position laid down by his predecessor, this restriction is substantially lifted when the law undergoes amendment. There is considerable criticism that Consumer Fora have not scrupulously followed the principle laid down by superior fora, that is State Commissions of the state and the National Commission and also that even superior fora have often not maintained settled positions laid down by their predecessors. The decisions of the National Commission and State Commissions are reported. However, there may not be uniformity with all such decisions. Furthermore, there may be conflicting decisions of various State Commissions and National Commissions. Consequently, while some legal experts have called for express provision to that effect, others feel that the principle being followed in respect of the Supreme Court and High Courts (since an appeal to Supreme Court is provided, High Courts are generally not expected to entertain consumer related writs though there is no such bar in the Act) may be generally followed even in respect of the decisions of State and National Commission subject to the interpretations if any of High Courts and the final legal position as laid down by Supreme Court.

### Clear case of medical negligence (similar to res ipsa loquitor?)

An appellant doctor was found by the State Commission to be responsible for leaving ribbon gauze in the right side of the nose after a septoplasty resulting in several complications. The complainant suffered and had to be under treatment all the while the National Commission confirmed the order and observed that it has no option but to deduce that it was a clear case of medical negligence on the part of the appellant. The National Commission in the case of Dr. Ravishankar *vs*. Jery K. Thomas and Anr, II (2006) CPJ 138 (NC) held that based on the facts and circumstances, the obvious deduction is that the appellant doctor is responsible for leaving behind ribbon gauze resulting in complications. Medical negligence was proved.

The brief facts of the case are as follows. The complainant was having some nasal and breathing problems. He approached the appellant doctor who upon examination advised a septoplasty, which was carried out on August 18, 1999 in second Respondent's hospital. It is the case of the complainant that after the operation, the pain aggravated and the breathing problem persisted. After examination, the complainant was advised to take some antibiotics for major nasal infection. Despite taking these medicines, the complainant was not getting any relief so he was taken to St. John's Hospital. A computed tomography (CT) scan showed that there was a deposit inside the nasal cavity for which an endoscopy was performed at St. John's hospital. Cotton gauze was removed from the nasal section on November 28, 2000. It was in these circumstances alleging medical negligence on the part of appellant and second respondent a complaint was filed before the State Commission. After hearing perusal of evidence and other material on record, the State Commission held the second respondent guilty of medical negligence and directed him to pay a compensation of Rs. 1 lac with interest @ 6% p.a from the date of complaint along with the cost of Rs. 5000/-. Aggrieved by this order, the Appellant doctor filed this appeal.

Held: heard the counsel for the appellant. As held by the State Commission, it is neither the surgery nor the procedure adopted that is under challenge. What is being challenged is the leaving behind of cotton gauze after surgery and the non removal of it by the appellant doctor. After going through the record maintained at St. John's hospital, Dr. Balasubramanium opined that after the CT scan a soft tissue mass (*gauze piece*) was found retained in the right nasal cavity that was removed under local anesthesia.

## CONCLUSION

In these circumstances, deduction is obvious that it was the appellant who was responsible for leaving behind ribbon gauze in the right side of the nose after the septoplasty performed by him on August 18, 1999 resulting in several complications. Because of this, the complainant suffered and had to be under treatment leaving us with no option but to deduce that it was a clear case of medical negligence on the part of the appellant.

## MEDICO LEGAL – SOME IMPORTANT ISSUES

The death of a patient while undergoing treatment does not amount to medical negligence.

In the case of Dr. Ganesh Prasad and Anr. V. Lal Janamajay Nath Shahdeo, I (2006) CPJ 117 (NC), the National Commission (Order: Per Mrs. Rajalaxmi Rao, Member) reiterated the principle that where proper treatment is given, death occurring due to process of disease and its complication, it can not be held that doctors and hospitals are negligent and orders of lower fora do not uphold the claim and award a compensation. In this case, a 4 ½ year old child suffering from cerebral malaria was admitted to the hospital. A life-saving injection was given. As opined by the child specialist, doses were safe and the treatment was proper. Though the death of the child is unfortunate, it can not be said that there was negligence on the part of the doctor.

The opinion based on teachings of one school of thought may not amount to medical negligence when there are two responsible schools of thought. Observations of the National Commission in the case of Dr. Subramanyam and Anr. *vs*. Dr. B. Krishna Rao and Anr., II (1996) CPJ 233 (NC) on the question of medical negligence are most illuminating as it involved a complaint by a well-qualified doctor against a fellow professional who treated his wife for an endoscopic sclerotherapy. It is relevant to note that in this case the complainant doctor alleged that the moment the patient was admitted to the Nursing Home, there was total mismanagement to the extent of virtually throwing her into the jaws of death solely because of negligence and improper rather wrong treatment given to her by the first opposite party, Dr. Rao. The complainants submitted that the slipshod, callous, and negligent way in which the patient was treated led to her death. Hon'ble Commission observed as follows: “The principles regarding medical negligence are well settled. A doctor can be held guilty of medical negligence only when he falls short of the standard of reasonable medical care. A doctor can not be found negligent merely because in a matter of opinion he made an error of judgment. It is also well settled that when there are genuinely two responsible schools of thought about management of a clinical situation the court could do no greater disservice to the community or advancement of medical science than to place the hallmark of legality upon one form of treatment.”

Error of judgment in diagnosis or failure to cure a disease does not necessarily mean medical negligence. In the case of Dr. Kunal Saha *vs*. Dr. Sukumar Mukherjee and Ors. III (2006) CPJ 142 (NC), the National Commission (per Mr. Justice M. B. Shah, President) considered the question of whether the Opponent doctors and hospital acted negligently in diagnosis of the disease suffered by the patient (wife of complainant doctor), administration of medicine (it was alleged that an overdose of steroids was prescribed), provision of facilities in hospital (absence of burn unit in hospital was alleged). A compensation of Rs. 77,76,73,500/- was claimed. The National Commission held that an error in medical diagnosis does not amount to deficiency in service. The National Commission further observed that the deceased (wife of Complainant) suffered from TEN (Toxic Epidermal Necrolysis), which is a rare disease and the mortality rate varies from 25% to 70% as per medical literature. The Commission also observed that considering the facts and circumstances of this case, the doctor can not be held liable for want of an exact diagnosis.

Role of expert opinion: in the case of Sethuraman Subramniam *Iyer vs*. Triveni Nursing Home and Anr. I (1998) CPJ 110 (NC), the National Commission dismissed the complaint holding that there was no expert evidence on behalf of the complainant. Similarly, in ABGP *vs*. Jog Hospital, the complaint was held to be not maintainable. In Farangi lal *Mutneja vs*. Shri Guru Harkishan Sahib Eye Hospital Sahana and Anr., IV (2006) CPJ 96, Union Territory Commission, Chandigarh dismissed the claim based on medical negligence with following observation: “The O.P. conducted an eye operation upon the complainant. The cornea was damaged subsequently, and visibility was lost. The complainant alleged that proper dilation of an eye was not done before conducting the cataract operation. Also it was alleged that the operation was done in a hurried manner. The Medical Council of India, after obtaining the expert opinion of two well known institutions, came to the conclusion that standard treatment protocol was followed and optimal procedures were carried out. Thus there was no negligence on the part of the O.P.”

Medical Literature: In the case of P. Venkata Lakshmi *vs*. Dr. Y. Savita Devi, II (2004) CPJ 14 (NC), the National Commission held that the State Commission ought to have considered the medical literature filed by the complainant and the State Commission had dismissed the complaint on the grounds that there was no expert evidence and remanded the matter.

Quantum of compensation: With regard to the quantum of compensation payable to an injured patient, the Supreme Court observed in the case of IMA *vs*. V.P. Shanta and Ors. III (1995) CPJ I (SC), as follows: “A patient who has been injured by an act of medical negligence has suffered in a way which is recognized by the law – and by the public at large as deserving compensation. This loss may be continuing and what may seem like an unduly large award may be little more than that sum which is required to compensate him for such matters as loss of future earnings and the future cost of medical or nursing care. To deny a legitimate claim or to restrict arbitrarily the size of an award would amount to substantial injustice. After all, there is no difference in legal theory between the plaintiff injured through medical negligence and the plaintiff injured in an industrial or motor accident.”

Engaging a specialist when available is obligatory. In the case of Prashanth S. *Dhananka vs*. Nizam Institute of Medical Science and Ors (1999) CPJ43 (NC), the National Commission deliberated on important issues such as what constitutes medical negligence, the duty of a hospital to engage a specialist when a specialist is available, vicarious liability of a hospital for omissions and commissions of doctors and staff, and compensation for mental and physical torture.

The National Commission on the question of whether compensation has to be awarded when doctors decide not to operate and the patient later dies. In the case of Narasimha Reddy and Ors. *Vs*. Rohini Hospital and Anr. I (2006) CPJ144 (NC), the National Commission held that when a patient could not be operated due to a critical condition, the doctor can not be held guilty of negligence if the proper course of practice is adopted and reasonable care is taken in administration of treatment. Consequently the Revision petition filed by the complainant was dismissed.

When a patient does not give a proper medical history, the doctor can not be blamed for the consequences. In the case of S. Tiwari *vs*. Dr. Pranav 1(1996) CPJ 301 (NC), it was alleged that a tooth was extracted without a proper test. When bleeding continued, the doctor administered a pain killer. Though the patient had a blood pressure of 130/90, he did not give the doctor his proper medical history. The National Commission upheld the findings of the State Commission and dismissed the complaint on the ground that the patient did not give a correct case history and follow-up when required.

Hospital is vicariously liable for any wrong claiming on the part of consultants. In the case of Ms Neha Kumari and Anr. V Apollo Hospital and Ors. 1 (2003) CPJ 145 (NC), the National Commission held that alleged medical negligence is not proved as the complainant suffered from complex birth defects of the spine and whole body as evidenced by a pre-operative CT scan. Two complaints were filed claiming a compensation of Rs. 26,90,000 alleging that while performing an operation (surgery) on the spinal canal, a rod was fitted inappropriately at the wrong level that resulted in the non functioning of the lower limbs. The Hon'ble commission held as follows:

“We do not find it is a case of medical negligence as alleged. Complaints have not denied that Neha Kumari was suffering from ailments from the very birth and that she was operated upon when she was only four years of age. On detailed investigations Neha Kumari was found to have multiple congenital complicated problems in Kiphoscoliotic deformity with weakness and wasting right upper limbs and (i) complex Khyphoscoliotic deformity of the mid dorsal spine with hemivertibrae of the D and D6 spinal levels and spinal bifida of the D and D7 vertebrae….Further filing of the appeal was delayed and no sufficient cause was shown to the satisfaction of Commission.

However, on the question of vicarious liability of the hospital for negligence on the part of the consultants, the Hon'ble Commission relying on the judgment in Basant Seth V Regency Hospital O P No.99 of 1994 rejected the contention of the hospital and held that the hospital is vicariously liable for any wrong claiming on the part of consultants.

Award of ex-gratia compensation against doctors and hospitals is not proper. The decision of the Supreme Court in the State of Punjab *vs*. Shiv Ram and Ors., IV (2005) CPJ 14 (SC) on a complaint alleging an unsuccessful family planning operation due to negligence of the doctor can be said to be an important milestone for many reasons. Firstly, the Supreme Court held that medical men and hospitals should not be saddled with damages unless they are found negligent. The apex court felt that awarding ex gratia compensation against doctors and hospitals without any findings on negligence is not proper. The court further held that there is a need for developing a welfare fund or insurance scheme. Failure of sterilization performed successfully is attributable to causes other than medical negligence and that the state government should think of devising and making provisions for a welfare fund or collaborating with insurance companies.

This judgment makes very pragmatic observations in the midst of several verdicts against medical professionals and hospitals especially when an award is made based on sympathetic considerations. It is heartening to note that the apex court looks at the issues relating to the medical profession and medical negligence in a holistic manner and with utmost consideration.

In a full bench decision dated August 25, 2005, Mr. Justice R.C. Lahoti, former C.J.I observed as follows: “Medical profession is one of the oldest professions of the world and is the most humanitarian one. There is no better service than to serve the suffering, wounded, and the sick. Inherent in the concept of any profession is a code of conduct, containing the basic ethics that underline the moral values that govern the professional practice and is aimed at upholding its dignity. Medical ethics underlines the values at the heart of the practitioner-client relationship. In the recent times, professionals are developing a tendency to forget that the self regulation which is at the heart of their profession is a privilege and not a right and the profession obtains this privilege in return for an implicit contract with society to provide good, competent and accountable service to the public. It must always be kept in mind that a doctor is a noble profession and the aim must be to serve humanity, otherwise the dignified profession will lose its true worth.”

The apex court further held that merely because a woman having undergone a sterilization operation became pregnant and delivered a child, the operating surgeon or his employer can not be held liable for payment of compensation on account of unwanted pregnancy or child. A claim in tort is sustainable only if there was negligence on the part of surgeon in performance of a surgery or the surgeon assured 100% exclusion of pregnancy after surgery. Proof of negligence will have to satisfy Bolam's test. Cause of failure of the sterilization operation may be obtained from laparoscopic inspection of the uterine tubes, by an X-ray examination, or by a pathological examination of the material removed at a subsequent operation of re-sterilization. The cause of action in the failed sterilization operation arises on account of negligence of the surgeon and not on account of child birth-failure due to natural causes.

The apex court reaffirmed the above observations in the State of Haryana and Ors. *vs*. Raj Rani IV (2005) CPJ28 (SC) and held as follows: “Doctors can be held liable only in cases where failure of operation is attributable to his negligence and not otherwise. Medical negligence recognized percentage of failure of sterilization operation due to natural causes depending on techniques chosen for performing surgery. The pregnancy can be for reasons de hors any negligence of the surgeon. A fallopian tube that is cut and sealed may reunite and the woman may conceive though a surgery is performed. Neither can the surgeons can be held liable to pay compensation nor can the state be held vicariously liable in such cases. However, payment made by the state will be held as ex gratia payment and the money paid to the poor will not be recovered.”

